# SMCHD1 Is Dispensable for Repeat-Induced FMR1 Hypermethylation in Fragile X Pluripotent Stem Cells

**DOI:** 10.3390/ijms27167224

**Published:** 2026-08-13

**Authors:** Uria Aviel, Adi Kababw-Florentin, Manar Abu Diab, Yotam Drier, Silvina Epsztejn-Litman, Rachel Eiges

**Affiliations:** 1 Stem Cell Research Laboratory, Medical Genetics Institute, Shaare Zedek Medical Center, Jerusalem 9112102, Israel or uria.aviel@mail.huji.ac.il (U.A.); adi.kababw@mail.huji.ac (A.K.-F.); manar.abudiab@mail.huji.ac.il (M.A.D.); silviel@szmc.org.il (S.E.-L.); 2 Faculty of Medicine, The Hebrew University of Jerusalem, Jerusalem 9112102, Israel; yotam.drier@mail.huji.ac.il; 3 The Lautenberg Center for Immunology and Cancer Research, The Institute for Medical Research Israel-Canada, The Hebrew University of Jerusalem, Jerusalem 9112102, Israel

**Keywords:** fragile X syndrome, SMCHD1, FMR1 DNA methylation, pluripotent stem cells

## Abstract

Fragile X syndrome (FRAX) is caused by CGG repeat expansion in the *FMR1* gene, which triggers aberrant DNA hypermethylation, chromatin condensation, and transcriptional gene silencing. However, the mechanism that underlies this repeat-induced epigenetic defect remains poorly understood. SMCHD1 is a chromatin regulator that promotes de novo DNA methylation and heterochromatin formation at long repetitive elements, including the D4Z4 macrosatellite repeat implicated in facioscapulohumeral muscular dystrophy (FSHD). Given the mechanistic parallels between FSHD and FRAX, we hypothesized that SMCHD1 contributes to repeat-induced *FMR1* hypermethylation. To test this, we disrupted *SMCHD1* in an XY FRAX human embryonic stem cell (hESC) line carrying a heavily methylated CGG-expanded *FMR1* allele. Despite efficient loss of *SMCHD1*, *FMR1* hypermethylation remained unchanged, indicating that SMCHD1 is dispensable for *FMR1* gene silencing. We next combined *SMCHD1* knockout with CRISPR-mediated CGG repeat contraction to determine whether removal of the pathogenic mutation could restore the aberrant methylation. Nevertheless, *FMR1* hypermethylation was preserved in all edited clones. These findings demonstrate that, unlike D4Z4 silencing in FSHD, *FMR1* repeat-induced hypermethylation does not depend on SMCHD1 activity. Moreover, correction of the underlying CGG expansion through repeat contraction is insufficient to restore the normal hypomethylated state of the *FMR1* locus in pluripotent stem cells.

## 1. Introduction

Unstable noncoding repeat disorders such as fragile X syndrome (FRAX), myotonic dystrophy type 1 (DM1), and C9orf72-associated ALS/FTD (C9-ALS/FTD) are frequently associated with DNA hypermethylation and a shift from active to repressive histone modifications [1,2,3,4,5,6,7,8,9,10,11,12,13]. These epigenetic changes typically arise once the repeat tract exceeds a critical threshold, ultimately leading to local chromatin condensation. Although the pathological consequences differ among diseases, the upstream molecular mechanisms underlying repeat-induced epigenetic silencing are widely believed to be shared.

Among these disorders, FRAX is the most common inherited cause of intellectual disability and autism spectrum disorder. It is caused by expansion of a CGG trinucleotide repeat within the 5′ untranslated region of the *FMR1* gene on chromosome Xq27.3 [14]. When the repeat expands beyond approximately 200 copies (the full mutation), it undergoes extensive DNA hypermethylation and heterochromatin formation, resulting in transcriptional silencing of *FMR1* and loss of its encoded protein, FMRP [14,15,16,17]. Because FRAX is inherited as an X-linked disorder, affected males generally exhibit more severe clinical manifestations than heterozygous females, whose phenotype is partially mitigated by X-chromosome inactivation [15,16,17].

Substantial effort has been devoted to unravelling the molecular mechanisms by which unstable microsatellite expansions induce DNA hypermethylation, particularly at the *FMR1* locus. Proposed mediators include double-stranded RNAs, R-loops, and mismatch repair proteins; however, none has been conclusively validated (see review by [18]). In addition, some propose that atypical secondary structures formed by the repeat sequences directly interact with the de novo and maintenance methylating enzymes (DNMT3A/B and DNMT1, respectively), whereas others suggest that such structures aberrantly recruit chromatin re-modelers, ultimately leading to chromatin compaction.

The timing of DNA methylation acquisition in FRAX also remains unresolved (see review by [19]). While some studies indicate that methylation is established prior to fertilization, others suggest that it is developmentally regulated and dynamically maintained [20,21,22,23,24,25]. Importantly, the underlying mechanism must be sequence-specific, as the presence of the mutation (i.e., expanded CGG repeat array) is essential for triggering abnormal epigenetic modifications.

To elucidate the mechanism responsible for aberrant DNA methylation at full-mutation *FMR1* alleles in FRAX, we focused on chromatin modifiers known to regulate de novo methylation at long repetitive elements. Among these, SMCHD1 emerged as a compelling candidate. SMCHD1 plays a well-established role in facilitating de novo methylation and heterochromatin induction during early embryonic development [26,27,28]. A particularly relevant example is the D4Z4 macrosatellite repeat at chromosome 4q35, which is implicated in facioscapulohumeral muscular dystrophy (FSHD; OMIM #158901). Normally, the D4Z4 array is long, heavily methylated and has a compacted chromatin structure, thereby ensuring transcriptional silencing. When the chromatin becomes relaxed on the background of a permissive haplotype, the *DUX4* retrogene (embedded within the distal D4Z4 unit) becomes aberrantly de-repressed [29,30,31,32,33]. Ectopic *DUX4* expression is toxic in skeletal muscle cells, and through this drives FSHD pathology [34,35,36].

There are two genetic forms of FSHD (type 1 and 2). Both are characterized by hypomethylation of the D4Z4 repeat array and reduced binding of SMCHD1 at the locus. In FSHD type 1, hypomethylation is caused by contraction of the D4Z4 array to 1–10 repeat units [31]. In FSHD type 2, loss-of-function mutations in SMCHD1 (haplo-insufficiency), co-segregating with a moderately shortened D4Z4 array (typically 11–20 repeat units and, in rare cases, up to 40 repeats), disrupt the maintenance of D4Z4 repression [37,38,39].

Given SMCHD1’s preference for long repetitive sequences and its repeat size-dependent role in de novo methylation at D4Z4, particularly during early embryonic development, we hypothesized that SMCHD1 may similarly contribute to repeat-associated epigenetic silencing at the *FMR1* gene in FRAX. This possibility is supported by the parallels between FSHD and FRAX, in which expanded repeats trigger repressive epigenetic changes, including local DNA hypermethylation, H3K9me3 deposition, and subsequent heterochromatin formation.

We therefore asked whether SMCHD1 contributes to de novo methylation at the *FMR1* locus once CGG repeats reach the pathological threshold associated with FRAX, particularly in the pluripotent state, when de novo DNMT activity is highly active. The aim of this study, therefore, was to determine whether SMCHD1 is involved in establishing or maintaining aberrant methylation at the CGG-expanded *FMR1* locus in FRAX-affected hESCs (FXS hESCs).

## 2. Results

To assess whether SMCHD1 facilitates aberrant methylation at the *FMR1* gene in FXS hESCs, we first addressed the noticeable variability in aberrant methylation observed both within and across mutant hESC lines [40]. We selected an XY FXS hESC line with relatively high baseline *FMR1* methylation (SZ-FX6) and manually subcloned it to obtain a well-defined homogeneous starting cell population that carries a full mutation that is completely methylated (>95%) and epigenetically silenced [40].

**Figure 1:**
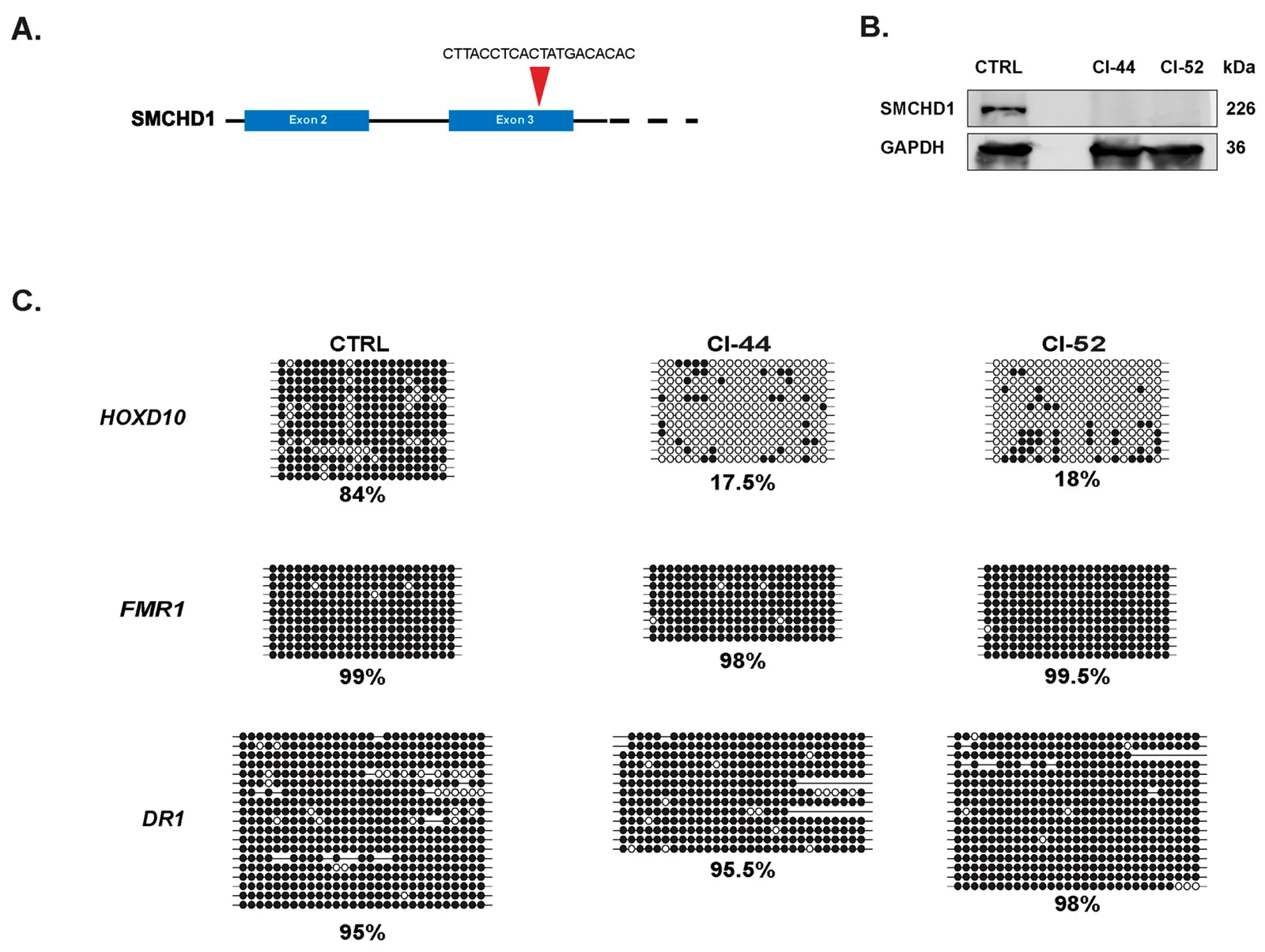
**Targeting *SMCHD1* in FXS hESCs harboring a heavily methylated *FMR1* CGG expansion.** (**A**) Schematic illustration of the CRISPR/Cas9-mediated strategy used to generate SMCHD1 knockout clones in FXS hESCs. (**B**) Western blot analysis confirming loss of SMCHD1 protein expression in two independent SMCHD1-knockout clones (Cl-44 and Cl-52) compared with isogenic FXS hESCs prior to gene editing (Control). GAPDH was used as a loading control. (**C**) Bisulfite sequencing analysis of DNA methylation patterns at the differentially methylated regions of *HOXD10* (top panel; 20 CpG sites), *FMR1* (middle panel; 22 CpG sites), and the D4Z4 repeat array (DR1 region; bottom panel; 29 CpG sites) in SMCHD1-knockout clones (Cl-44 and Cl-52) compared with the SMCHD1 wild-type FXS hESC control (CTRL). Methylation levels (%) are indicated below each clone. Each row represents an individual DNA molecule; open circles represent unmethylated CpG sites, and filled circles represent methylated CpG sites.

Next, we generated *SMCHD1* knockout hESC lines on the background of CGG-expanded and hypermethylated *FMR1* alleles by targeting exon 3 with a single gRNA using CRISPR/Cas9 editing (Figure 1A). Western blot analysis confirmed complete loss of SMCHD1 expression in two independent FXS hESC clones (Cl-44 and Cl-52) (Figure 1B).

First, to validate the functional consequence of SMCHD1 loss on DNA methylation in the pluripotent state, we examined methylation levels by bisulfite colony sequencing at the *HOXD10* locus, a known downstream target of SMCHD1 within the *HOX* gene cluster [41,42] (Figure 1C). As expected, SMCHD1 deficiency resulted in a robust reduction in *HOXD10* methylation (from 84% to less than 20%), confirming the loss of SMCHD1-dependent methylation activity.

We then assessed the methylation status of the mutant *FMR1* gene, focusing on the disease-associated differentially methylated region (DMR, 22CpG sites [40]). Despite complete SMCHD1 depletion, both FXS hESC clones remained fully *FMR1* hypermethylated (>95%, Figure 1C). These findings suggest that SMCHD1 is either not required for *FMR1* methylation, or that its loss alone is insufficient to overcome the heterochromatin state once it has been established at the mutant locus.

Nevertheless, because methylation at the D4Z4 array, which is a direct target of SMCHD1, was unaffected by *SMCHD1* knockout in the FXS hESCs (consistent with that the hESC line has a normal size D4Z4 alleles being derived from an embryo unaffected by FSHD) (≥95%, Figure 1C), we hypothesized that partial shortening of the CGG expansion might be necessary to reveal the impact of SMCHD1 loss. This possibility is consistent with previous studies showing that SMCHD1 deficiency alone is insufficient to induce D4Z4 hypomethylation and *DUX4* de-repression when the repeat array is long [38,39].

**Figure 2:**
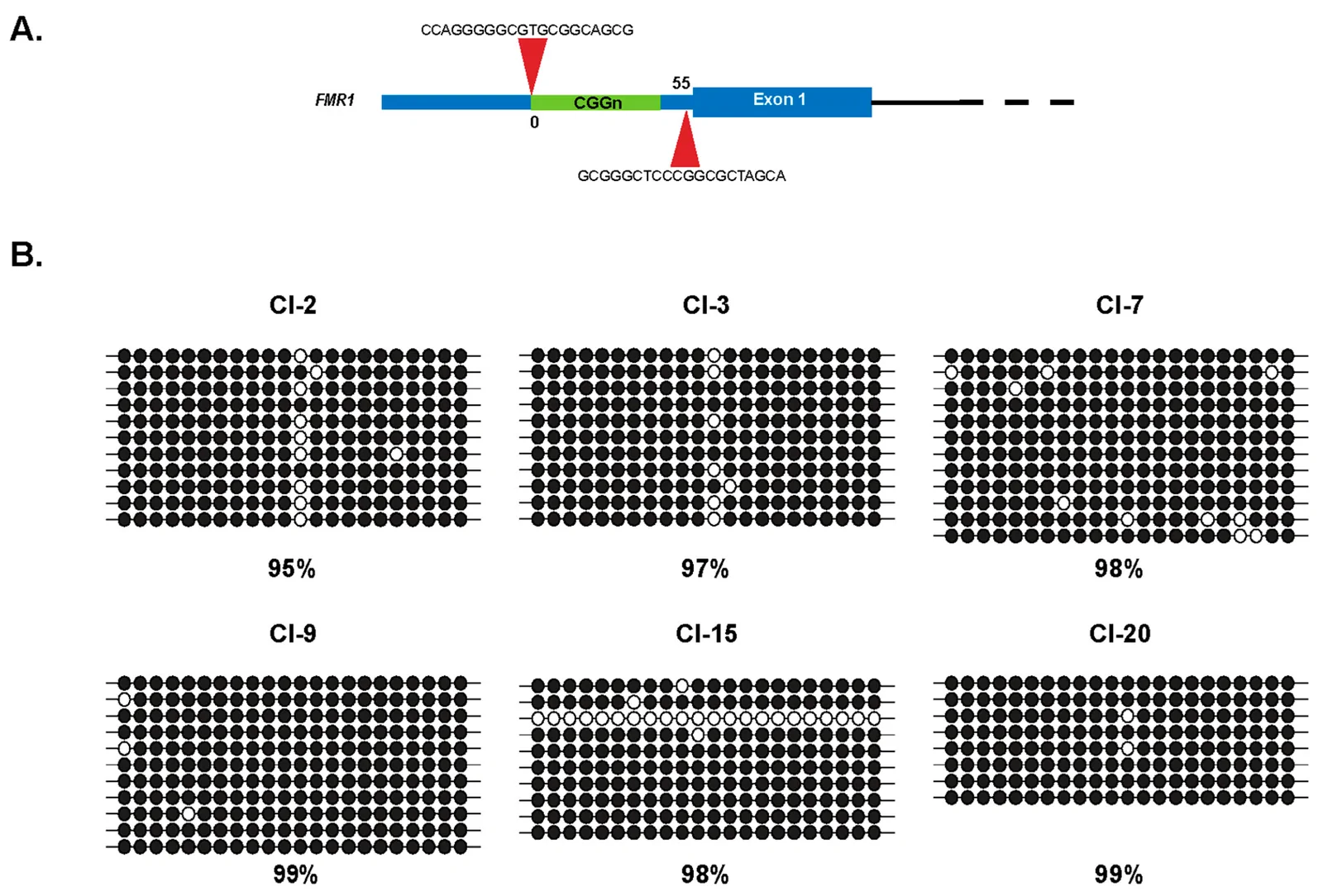
**Targeting the expanded CGG repeat tract in FXS hESCs harboring a heavily methylated FMR1 allele.** (**A**) Schematic illustration of the CRISPR/Cas9-mediated strategy used for excision/contraction of the expanded FMR1 CGG tract. (**B**) Bisulfite sequencing analysis of DNA methylation patterns at the *FMR1* differentially methylated region (22 CpG sites) following CGG repeat contraction in six different clones. Methylation levels (%) are indicated below each gene-edited clone. Each row represents an individual DNA molecule; open circles represent unmethylated CpG sites, and filled circles represent methylated CpG sites.

In light of these findings, and to better mimic the underlying mechanism of FSHD2, in which SMCHD1 haploinsufficiency affects moderately contracted repeat arrays, we first shortened the CGG expansion and subsequently investigated the impact of SMCHD1 deficiency on the methylation status of *FMR1* in the XY FXS hESCs. To achieve this, we targeted the CGG repeat expansion using two flanking gRNAs simultaneously, with the PAM sequences positioned in the first CGG unit and 55 bp downstream of the repeat tract (Figure 2A). Successful repeat excision was confirmed by PCR, GeneScan analysis, and Sanger sequencing (Figures S1 and S2). Gene editing generated at least six clones displaying contraction of the CGG repeat tract into the normal range (CGG10 [Cl-2], CGG12 [Cl-3], CGG10 [Cl-7], CGG19 [Cl-9], CGG11 [Cl-15] and CGG12 [Cl-20]) (Table S2 and Figure S2). Strikingly, despite contraction of the CGG array into the normal range, all CGG-contracted FXS hESC clones remained fully methylated at the disease-associated DMR (≥89%, Figure 2B).

**Figure 3:**
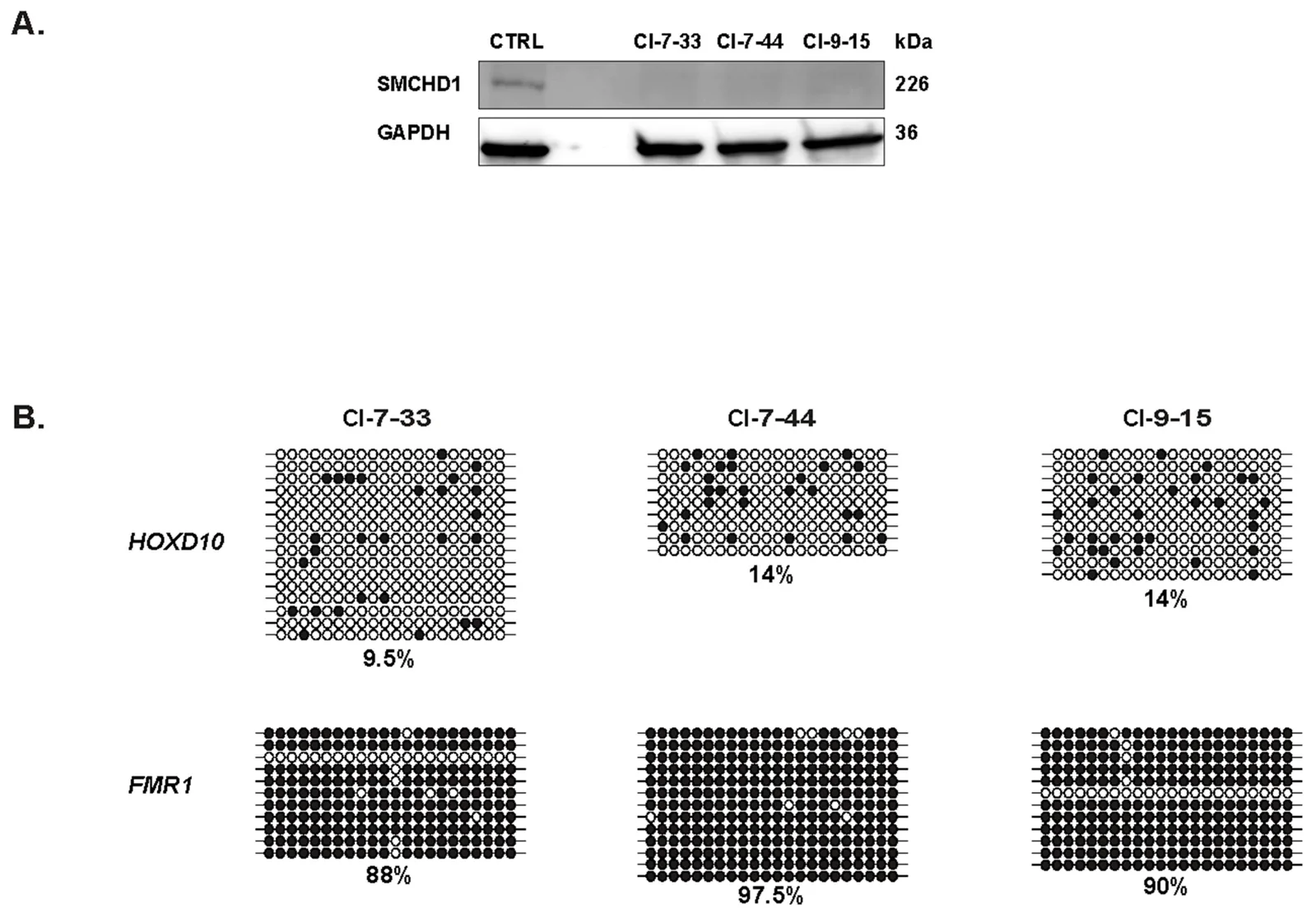
**Targeting *SMCHD1* in FXS hESCs carrying a hypermethylated CGG-contracted *FMR1* allele.** (**A**) Western blot analysis confirming loss of protein expression following *SMCHD1* gene targeting in FXS hESCs after CGG contraction. Shown, three independent edited clones (Cl-7-33, Cl-7-44, and Cl-9-15) and their isogenic control, unmanipulated FXS hESCs (CTRL). GAPDH was used as a loading control. (**B**) Bisulfite sequencing analysis of DNA methylation patterns at the differentially methylated regions of *HOXD10* (top panel; 20 CpG sites) and *FMR1* (bottom panel; 22 CpG sites) following combined targeting of the CGG repeats and SMCHD1 expression in three independent FXS hESC clones. Methylation levels (%) are indicated below each gene-edited clone. Each row represents an individual DNA molecule; open circles represent unmethylated CpG sites, and filled circles represent methylated CpG sites.

Next, to mimic the pathogenic mechanism of SMCHD1 in FSHD2, where SMCHD1 haploinsufficiency acts on at least moderately contracted D4Z4 repeats to induce hypomethylation and chromatin relaxation [37,43], we disrupted *SMCHD1* in the background of hypermethylated, CGG-contracted XY FXS hESC clones. Western blot analysis confirmed complete loss of SMCHD1 expression (Figure 3A). Consistent with this, bisulfite colony sequencing revealed a dramatic reduction in DNA methylation at the *HOXD10* locus (to less than 15%), confirming the loss of SMCHD1 function in promoting DNA methylation (Figure 3B).

We subsequently re-examined methylation at the *FMR1* DMR in the hypermethylated CGG-contracted clones after the knockout of *SMCHD1*. However, despite CGG contraction into the normal range, none of the analyzed clones (*n* = 3) exhibited a reduction in *FMR1* methylation levels (≥88%, Figure 3B). These findings suggest that, despite apparent similarities in the epigenetic silencing mechanisms associated with the *FMR1* and D4Z4 repeat elements, SMCHD1 does not play a role in maintaining abnormal methylation at the *FMR1* gene, not even when the CGG array is contracted. Alternatively, they raise the possibility that *FMR1* hypermethylation becomes irreversible once established, even following correction of the underlying repeat expansion.

Collectively, these findings demonstrate that (1) contraction of the CGG repeat expansion into the normal range in XY FXS hESCs is insufficient to reverse *FMR1* hypermethylation, and that (2) unlike in FSHD2, SMCHD1 does not appear to contribute at the pluripotent state to DNA methylation at the *FMR1* locus in FRAX.

## 3. Discussion

Given the striking molecular parallels between facioscapulohumeral muscular dystrophy (FSHD) and fragile X syndrome (FRAX), particularly the strong association between expanded repetitive elements, DNA hypermethylation, chromatin compaction and epigenetic gene silencing, we investigated whether SMCHD1 might similarly contribute to hypermethylation at the *FMR1* gene in FRAX.

In FSHD, SMCHD1 plays a central role in maintaining D4Z4 repeat-associated heterochromatin, and its dysfunction leads to chromatin relaxation and de-repression of the pathogenic *DUX4* retro-element in patient muscle cells [37,44,45]. Furthermore, SMCHD1 has been shown to associate with additional CG-rich repetitive elements, including long telomeric microsatellite regions [46], suggesting a broader role in regulating repeat-associated chromatin architecture. These observations raised the possibility that SMCHD1 may also participate in the epigenetic silencing of the expanded CGG repeat at *FMR1* in FRAX patients. We hypothesized that, analogous to FSHD, where neither moderate repeat contraction nor SMCHD1 haploinsufficiency alone is sufficient to induce chromatin relaxation and disease manifestation, the contribution of SMCHD1 to *FMR1* silencing might only become evident when both independent molecular events are combined. To test this possibility, we generated *SMCHD1* knockout hESC lines in both CGG-expanded and CGG-corrected fragile X backgrounds. However, loss of SMCHD1 had no effect on DNA methylation levels at the DMRs of the mutant *FMR1* locus.

Together, these findings suggest that the mechanisms underlying *FMR1* hypermethylation and gene silencing in FRAX differ from those governing methylation at the D4Z4 macrosatellite. This distinction may, at least in part, be attributed to a difference in the genomic architecture of these loci. Whereas D4Z4 is a large subtelomeric macrosatellite composed of tandem repeat units that collectively span tens of kilobases and organize into higher-order chromatin domains, the pathogenic *FMR1* mutation consists of a comparably short CGG microsatellite expansion, ranging from a few hundred to a few thousand base pairs in length, located within the 5′ UTR of a single-copy gene.

An important limitation of the present study, however, is that our experimental design examines the maintenance, rather than the establishment, of *FMR1* hypermethylation, as the parental hESC line already harbored a fully methylated *FMR1* allele. Thus, although our findings demonstrate that SMCHD1 is dispensable for maintaining the methylated state in pluripotent cells, they do not exclude a role for SMCHD1 during the initial step of *FMR1* methylation in early embryonic development. Indeed, previous studies have shown that SMCHD1 functions primarily during the establishment of de novo DNA methylation at both the D4Z4 macrosatellite and the inactive X chromosome, whereas its deletion after these epigenetic states have been established has only a modest impact on DNA methylation [28,30,47,48]. It is therefore conceivable that *FMR1* hypermethylation is likewise established during a restricted developmental window preceding the pluripotent state, after which it becomes stably maintained and refractory to reversal, even following removal of the initiating trigger, the pathogenic CGG repeat expansion.

On the other hand, the contribution of SMCHD1 to *FMR1* silencing may only become apparent when DNA methylation is also disrupted. Consistent with this possibility, Wang and colleagues showed that in mouse embryonic fibroblasts, neither *SMCHD1* knockout nor treatment with the DNA demethylating agent 5-aza-2′-deoxycytidine were sufficient in the reactivation of genes on the inactive X chromosome. However, their combined effect induced robust gene reactivation, indicating that these pathways cooperate to maintain stable epigenetic silencing [47]. Future studies will be required to determine whether *FMR1* is regulated by a similar mechanism, whether SMCHD1 is required primarily for the establishment rather than the maintenance of *FMR1* hypermethylation, or whether maintenance of the silent state is largely independent of SMCHD1.

One important observation from this study is that CGG repeat contraction alone is insufficient to restore the normal epigenetic state of the *FMR1* locus in FXS hESCs. Previous studies have reported variable outcomes following repeat correction. Park et al. used CRISPR/Cas9 to remove the CGG expansion from FXS iPSCs using a single gRNA targeting the repeat region [49]. This approach resulted in imprecise deletions spanning the repeat tract and approximately 100 bp of flanking sequence. In the successfully edited clone, repeat removal was accompanied by loss of promoter hypermethylation, chromatin relaxation, and restoration of *FMR1* expression to wild-type levels.

In contrast, Xie et al. used paired gRNAs targeting sequences immediately flanking the CGG expansion, enabling more precise excision [50]. Although repeat removal restored *FMR1* expression and promoter demethylation in somatic cell hybrids, epigenetic reprogramming occurred in only 20% (1/5) of edited FXS iPSCs, indicating that repeat excision alone is generally insufficient to reverse the established epigenetic defects.

The discrepancy between the original report by Park et al. and our study, as well as the findings of Xie and colleagues, may be explained by differences in editing strategies and experimental design. Furthermore, our conclusions are based on multiple independent edited clones rather than a single clone. Together, these findings suggest that, once established, *FMR1*-associated epigenetic silencing may become uncoupled from the initiating CGG expansion event.

In contrast to *FMR1*, excision of the CTG expansion at the *DMPK* locus in congenital myotonic dystrophy type 1 (CDM1) cells produced a markedly different outcome [51]. Removal of the expanded repeat (CTG2000 in the 3′ UTR of *DMPK*) in hESCs and iPSCs, but not differentiated cells, restored the normal epigenetic state of the locus, indicating that maintenance of aberrant chromatin in this context remains dependent on the continued presence of the mutation.

The distinct outcomes of repeat targeting at *DMPK* and *FMR1* may be explained by differences in repeat sequence composition (methylated CGGs vs. unmethylated CTGs), repeat location within the gene (5′ UTR vs. 3′ UTR), or differential recruitment and activity of de novo vs. maintenance DNA methyltransferases at these loci. Further studies will be required to define the molecular mechanisms underlying these locus-specific differences.

## 4. Materials and Methods

### 4.1. Cell Culture

The fragile X syndrome human embryonic stem cell (hESC) line SZ-FX6 (46,XY), derived from a preimplantation genetic diagnosis (PGD)-generated embryo, was established and used under protocols approved by the Institutional Review Board of Shaare Zedek Medical Center (IRB 87/07, 16 September 2009). Embryo donation was conducted in accordance with the regulations of the Israeli Ministry of Health National Ethics Committee and the guidelines of the NIH and ISSCR. All donated embryos were surplus from PGD procedures and would otherwise have been discarded. Informed consent was obtained under a strict separation of roles between the genetic counselor responsible for the consent process, the attending IVF physician, and the research team.

Human embryonic stem cell (hESC) line derivation and characterization were performed as previously described [52]. FRAX-affected hESCs (SZ-FX6 cell line carrying a CGG expansion of >200 repeat units; Avitzour et al. [39]) and corresponding gene-edited derivatives were grown either on Mitomycin C (Sigma-Aldrich M0503-5X2MG, St. Louis, MO, USA) treated mouse embryonic fibroblasts (MEFs) as a feeder using Knockout™ DMEM (Gibco™, Thermo Fisher Scientific, 10829-018, Waltham, MA, USA) as previously described in [53], or under feeder-free conditions in mTeSR Plus medium (STEM CELL™ TECHNOLOGIES, 100-0274, Vancouver, BC, Canada) on vitronectin-coated plates. Culture plates were coated with 9 μg/mL Vitronectin (VTN-N) recombinant Human protein, Truncated (Gibco™, Thermo Fisher Scientific, A400458, Waltham, MA, USA). Cells were seeded 1 h after coating. For passaging, cells were dissociated using Accutase (STEMCELL™ TECHNOLOGIES, 07922, Vancouver, BC, Canada) and replated in mTeSR Plus medium supplemented with 10 µM ROCK inhibitor (Y-27632; Cayman Chemical Company, 10005583, Ann Arbor, MI, USA).

### 4.2. Gene Editing by Electroporation

#### 4.2.1. gRNA Cloning

Guide RNAs (gRNAs) were designed to target exon 3 of the *SMCHD1* gene (5′-CTTACCTCACTATGACACAC-3′) or sequences flanking the CGG repeat region of the *FMR1* gene (upstream gRNA: 5′-CCAGGGGGCGTGCGGCAGCG-3′; downstream gRNA: 5′-GCGGGCTCCCGGCGCTAGCA-3′) using the Zhang laboratory CRISPR design web tool (crispr.mit.edu).

gRNAs were cloned into the pSpCas9(BB)-2A-Puro (PX459) V2.0 vectors (Addgene, 62988, Watertown, MA, USA) as previously described [51]. Briefly, complementary oligonucleotides containing the gRNA sequence and compatible BbsI overhangs were annealed and ligated into the linearized PX459 vector using T4 DNA ligase (New England Biolabs, M0202, Ipswich, MA, USA). Correct insertion of the gRNA sequences was verified by Sanger sequencing (Applied Biosystems 3130 Genetic Analyzer, Foster City, CA, USA) using Big Dye Terminator v1.1 Cycle sequencing Kit (Thermo Fisher Scientific, AB-4337450, Waltham, MA, USA) the U6 forward primer (5′-GAGGGCCTATTTCCCATGATTCC-3′).

#### 4.2.2. Electroporation of hESCs

Undifferentiated hESCs were maintained under feeder-free conditions in mTeSR Plus medium on 9 μg/mL Vitronectin (VTN-N) recombinant Human protein, Truncated (Gibco™, A400458, Thermo Fisher Scientific, Waltham, MA, USA). One day prior to electroporation, drug-resistant MEFs (DR3) were plated on 0.2% gelatine (Sigma-Aldrich, G1890, St. Louis, MO, USA) coated 6-well plate.

To generate clonal edited lines, 8 × 10^5^ hESCs were electroporated using Amaxa™ P3 primary cell 4D-Nucleofector™ X Kit L (Lonza Bioscience, V4XP-3024, Basel, Switzerland) with a 4D-Nucleofector™ core unit (Lonza Bioscience, AAF-1003B, Basel, Switzerland) with pSpCas9(BB)-2A-Puro (PX459) V2.0 plasmids expressing either a single gRNA targeting exon 3 of SMCHD1 or a pair of gRNAs targeting sequences flanking the FMR1 CGG repeat region. Cells were grown in mTeSR Plus medium, and twenty-four hours after electroporation were treated with puromycin (0.2 µg/mL) (Sigma-Aldrich, P8833, St. Louis, MO, USA) for 48 h. Mitomycin C-treated MEFs were subsequently added to support the expansion of individual edited clones. Puromycin-resistant colonies were manually isolated, expanded on feeder cells, and subsequently adapted to feeder-free culture conditions.

#### 4.2.3. Screening and Validation of Edited Clones

Successful targeting of the *SMCHD1* locus was initially assessed by screening for genome-editing-induced sequence alterations. Genomic DNA was extracted from a small number of cells from each clone and analyzed by Sanger sequencing using primers listed in Table S2. Clones carrying potential *SMCHD1* knockout (KO) alleles were further validated by assessing *SMCHD1* protein expression using Western blot analysis.

To identify deletions of the *FMR1* CGG repeat expansion, genomic DNA was extracted from individual clones and amplified by nested PCR using FastStart DNA polymerase (Roche, #04738357001, Basel, Switzerland) and primers flanking the repeat region (Table S2). PCR products were analyzed by GeneScan using an ABI 3130 Genetic Analyzer with a fluorescently labeled forward primer (5′-FAM primer: 5′-CAGCTCCTCCATCTTCTCTTCAG) and reverse primer (3′ primer: 5′-CTCAGCTCCGTTTCGGTTTC-3′). Clones showing reduced or absent CGG repeat expansion were further validated by Sanger sequencing to confirm repeat excision.

### 4.3. Western Blot Analysis

Following cell lysis in Tris–Triton lysis buffer supplemented with protease inhibitor cocktail (1:100 dilution) (Sigma-Aldrich, P8340-1ML, St. Louis, MO, USA), total protein concentration was determined using the Bradford assay (Thermo Scientific, 1856209, Waltham, MA, USA). Equal amounts of protein lysates were separated by SDS-PAGE using Mini-PROTEAN TGX precast gels (Bio-Rad, 4561094, Hercules, CA, USA) and transferred onto nitrocellulose membranes using the Trans-Blot^®^ Turbo™ Transfer System (Bio-Rad, 1704150, Hercules, CA, USA). Membranes were blocked and incubated with primary antibodies against SMCHD1 (1:2500) (rabbit antibody; Bethyl Laboratories, A302-872A, Montgomery, TX, USA) or the housekeeping protein GAPDH (1:2000) (mouse antibody; Abcam, ab8245, Cambridge, UK). Following incubation with appropriate HRP-conjugated secondary antibodies-goat anti-rabbit or goat anti-mouse (1:5000 and 1:10,000 respectively) (Abcam, ab6721 and ab205719, Cambridge, UK), protein signals were detected using a chemiluminescence-based detection method (Cyanagen, XLS071,0250, Bologna, Italy).

### 4.4. Locus-Specific Bisulfite Colony Sequencing

Genomic DNA (2 µg) was subjected to bisulfite conversion using the EZ DNA Methylation Kit (Zymo Research, D5021, Irvine, CA, USA) and according to manufacturer’s protocol. Bisulfite-treated DNA was amplified using EpiTaq HS DNA polymerase (Takara Bio, R007A, Kusatsu, Shiga, Japan). PCR products generated with bisulfite-converted primers designed to detect both methylated and unmethylated alleles were cloned into a pGEM^®^-T Easy Vector (Promega, A1360, Madison, WI, USA). Following transformation, individual colonies were analyzed for CpG methylation patterns by Sanger sequencing using T7 and SP6 primers (T7: 5-TAATACGACTCACTATAGGG-3, SP6: 5-ATTTATGACACTATAG).

## Data Availability

The original contributions presented in this study are included in the article/supplementary material. Further inquiries can be directed to the corresponding author.

## Supplementary Materials

The following supporting information can be downloaded at: https://www.mdpi.com/article/10.3390/ijms27167224/s1.
