# Supplementary material for: SMCHD1 Is Dispensable for Repeat-Induced FMR1 Hypermethylation in Fragile X Pluripotent Stem Cells
Source: Int J Mol Sci. 2026 Aug 13;27(16):7224. doi: 10.3390/ijms27167224 (PMC13513341; doi:10.3390/ijms27167224)

FIGURE S1

CI-2

CI-3

CI-7

CI-9

CI-15

CI-20

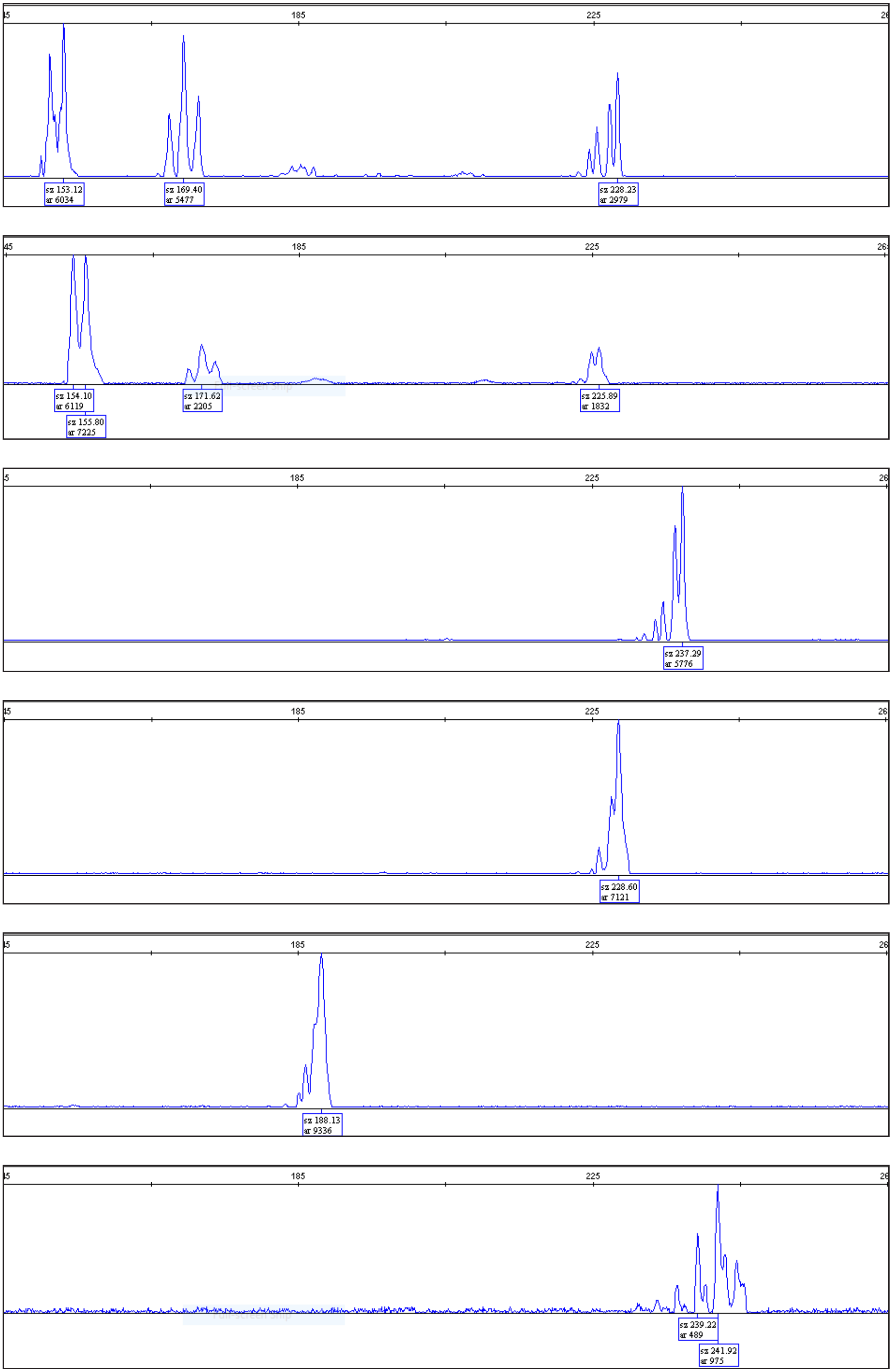

Supplement: Supplementary file 1 [file ijms-27-07224-s001.zip › Figure S1.pdf]
