# Supplementary material for: SMCHD1 Is Dispensable for Repeat-Induced FMR1 Hypermethylation in Fragile X Pluripotent Stem Cells
Source: Int J Mol Sci. 2026 Aug 13;27(16):7224. doi: 10.3390/ijms27167224 (PMC13513341; doi:10.3390/ijms27167224)
Supplement: Supplementary file 1 [file ijms-27-07224-s001.zip › Figure S2.pdf]

FIGURE S2

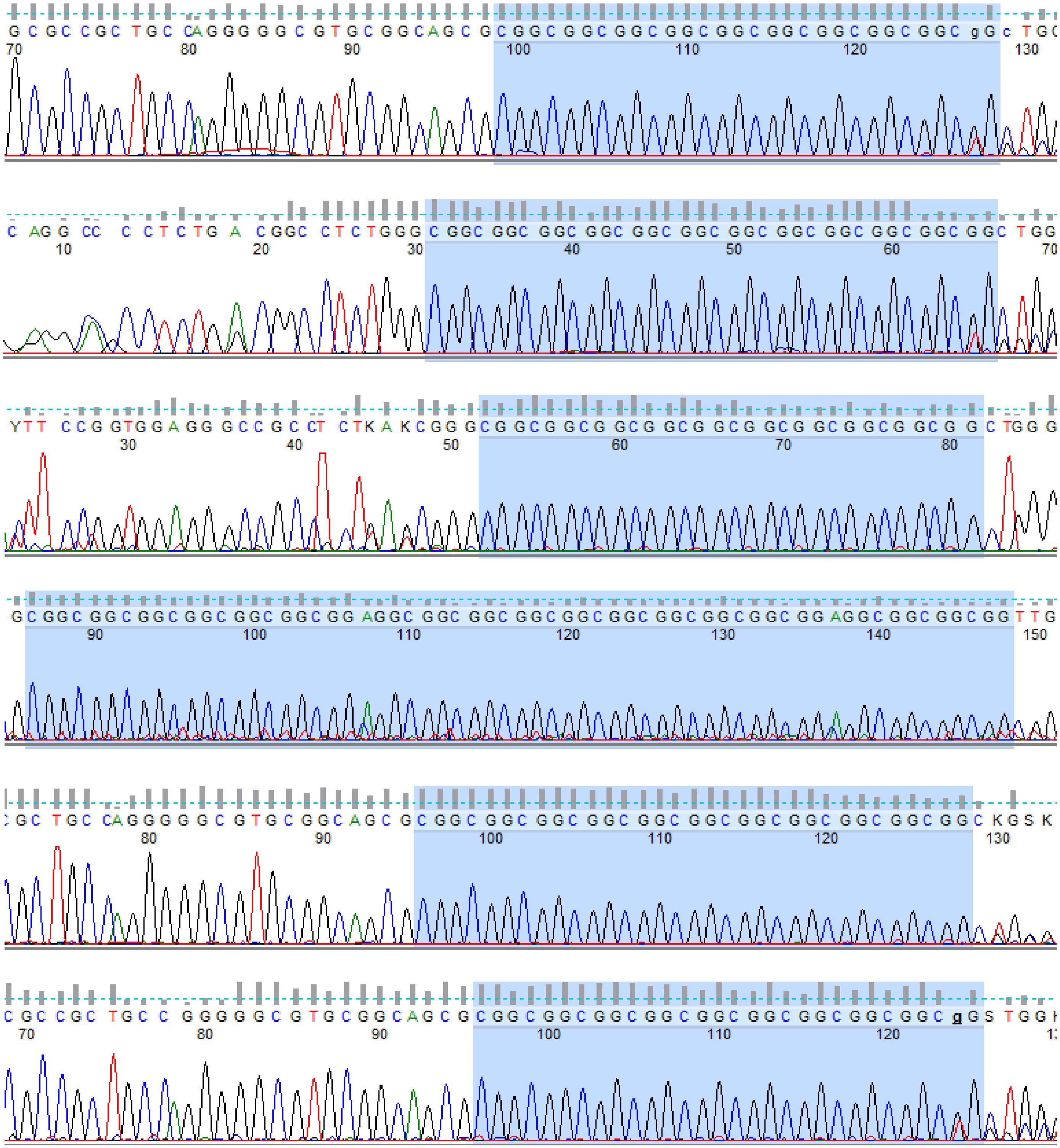

Cl-2

Cl-3

Cl-7

Cl-9

Cl-15

Cl-20
