# Supplementary material for: SMCHD1 Is Dispensable for Repeat-Induced FMR1 Hypermethylation in Fragile X Pluripotent Stem Cells
Source: Int J Mol Sci. 2026 Aug 13;27(16):7224. doi: 10.3390/ijms27167224 (PMC13513341; doi:10.3390/ijms27167224)
Supplement: Supplementary file 1 [file ijms-27-07224-s001.zip › Tabel S1.pdf]

Table S1. Summary of Sanger Sequencing Analysis for Residual CGG Units and Indel Mapping

Clone	Residual repeat units	Indels (relative to the nearest breakpoint)
2	10	none
3	12	67 bp deletion (upstream to the CGGs)
7	10	63 bp deletion (upstream to the CGGs)
9	19	2 bp deletion (downstream to the CGGs)
15	11	none
20	10	none
