# Supplementary material for: SMCHD1 Is Dispensable for Repeat-Induced FMR1 Hypermethylation in Fragile X Pluripotent Stem Cells
Source: Int J Mol Sci. 2026 Aug 13;27(16):7224. doi: 10.3390/ijms27167224 (PMC13513341; doi:10.3390/ijms27167224)
Supplement: Supplementary file 1 [file ijms-27-07224-s001.zip › Table S2.pdf]

Table S2. Primer Sequences and PCR Amplification Conditions

Locus	Locus	Primer set	Annealing temperature	Product size (bp)
Sanger sequencing	*SMCHD1* (KO validation)	F: 5-TCTTGATTACAGATGAAACT-3<br>R: 5-AAGGCAGGTAATAATTTATC-3	50	220
Sanger sequencing and GeneScan Analysis	*FMR1* (CGG contraction) 1ST round	F: 5-CGTGACGTGGTTTCAGTGTTTAC -3<br>R: 5-TGCCCTAGAGCCAAGTACCTTGTA-3	58	378
	FMR1 (CGG contraction) 2nd round	F: 5-CTCAGCTCCGTTTCGGTTTC-3<br>R: 5-CAGCTCCTCCATCTTCTCTTCAG-3	60	144
Bisulfite sequencing	*HOXD10*	F: 5-TAGTTGTAGATGGAAAAGAAGATGA-3<br>R: 5-TATCTCAAAACTAAAATCACCCC-3	58	317
	FMR1	F: 5-TTGAGTGTATTTTTGTAGAAATGGG-3<br>R: 5-CCTCTCTCTTCAAATAACCTAAAAA-3	60	191
	D4Z4 (DR1)	F: 5-GAAGGTAGGGAGGAAAAG-3<br>R: 5-ACTCAACCTAAAAATATACAATCT-3	54	253
